# Indications for free vascularized fibular grafting for the treatment of osteonecrosis of the femoral head

**DOI:** 10.1186/1471-2474-8-78

**Published:** 2007-08-08

**Authors:** Kenji Kawate, Hiroshi Yajima, Kazuya Sugimoto, Hiroshi Ono, Tetsuji Ohmura, Yasunori Kobata, Keiichi Murata, Koji Shigematsu, Kenji Kawamura, Ikuo Kawahara, Naoki Maegawa, Katsuya Tamai, Yoshinori Takakura, Susumu Tamai

**Affiliations:** 1 Department of Orthopaedic Surgery, Nara Medical University, Japan; 2 Department of Orthopaedic Surgery, Saiseikai Nara Hospital, Japan; 3 Department of Orthopaedic Surgery, Kokuho Central Hospital, Japan; 4 Department of Orthopaedic Surgery, Todaiji Seishien, Japan; 5 Department of Orthopaedic Surgery, Heisei Rehabilitation School, Japan

## Abstract

**Background:**

The present study aimed to determine the indications for free vascularized fibular grafting for the treatment of osteonecrosis of the femoral head.

**Methods:**

Seventy-one hips (60 patients) were clinically followed for a minimum of 3 years. Average follow-up period was 7 years. Etiologies were alcohol abuse in 31 hips, steroid use in 27, idiopathic in 7 and trauma in 6. Preoperative staging of the necrotic lesion was done using the Steinberg's classification system. The outcomes of free vascularized fibular grafting were determined clinically using the Harris hip-scoring system, radiographically by determining progression, and survivorship by lack of conversion to total hip replacement.

**Results:**

The average preoperative Harris hip score was 56 points and the average score at the latest follow-up examination was 78 points. Forty-seven hips (67%) were clinically rated good to excellent, 4 hips (6%) were rated fair, and 20 hips (28%) were rated poor. Thirty-six hips (51%) did not show radiographic progression while 35 hips (49%) did, and with an overall survivorship of 83% at 7 years. Steroid-induced osteonecrosis was significantly associated with poor scores and survival rate (68%). Preoperative collapse was significantly associated with poor scores, radiographic progression and poor survival rate (72%). A large extent of osteonecrosis greater than 300 degrees was significantly associated with poor scores, radiographic progression and poor survival rate (67%). There was no relationship between the distance from the tip of the grafted fibula to the subchondral bone of the femoral head and postoperative radiographic progression.

**Conclusion:**

In conclusion, small osteonecrosis (less than 300 degrees of the femoral head) without preoperative collapse (Steinberg's stages I and II) is the major indication for free vascularized fibular grafting. Steroid-induced osteonecrosis is a relative contraindication. Large osteonecrosis (greater than 300 degrees) with severe preoperative collapse (greater than 3 mm) is a major contraindication. Hips with 2 negative factors such as severe preoperative collapse and a large extent of osteonecrosis, require hip replacements.

## Background

Osteonecrosis of the femoral head in adults is still not well understood, and often affects young patients aged 20 to 50 years old [1]. Osteonecrosis initially causes death of bone which then collapses in certain instances and later may result in cartilage destruction if secondary degenerative changes develop [2,3]. Several procedures have been developed to prevent the conversion to total hip replacement especially in young patients, because total hip replacement in young patients is associated with a high rate of revised surgery [4-6].

Core decompression has demonstrated some success for the treatment of hips without preoperative collapse, but it has been less successful for hips that had already collapsed [7]. Varus [8] and rotational osteotomies [9] are effective for hips with preoperative collapse, but these technically demanding procedures have yielded mixed results that often are difficult to reproduce. Curettage of the lesion followed by bone grafting is thought to be insufficient for revascularization [10]. Although the vessel transplantation procedure reported by Hori et al. is a biological approach, it does not yield to sufficient biomechanical support [11]. Vascularized pedicle iliac bone grafting has been partially successful for the treatment of hips without preoperative collapse but it still does not yield to sufficient biomechanical support [12,13].

With regards to free vascularized fibular grafting, Yoo et al. reported excellent outcomes for the operation of 81 hips in 73 patients, with an average of 5 years and 2 months follow-up, in 1992 [14]. Since then, many researchers have reported the outcomes of free vascularized fibular grafting [15-19]. According to previous reports, the stage of the disease at the time of treatment is a major factor determining the success or failure of the surgical procedures that will preserve the femoral head [14-18]. Other factors that may be of prognostic importance include etiology, size of the necrotic lesion [17], and location of the fibula. The present study aimed to assess the outcomes of free vascularized fibular grafting including etiology, stage, extent of osteonecrosis, and location of the fibula; and determine the indications for this procedure.

## Methods

Between April 1992 and December 2003, 73 consecutive free vascularized fibular grafting procedures for the treatment of osteonecrosis of the hip were performed in 62 patients by the 6 authors (K.K, H.Y., H.O., T.O., Y.K., S.T.). The surgical team consisted of hip surgeons and microvascular surgeons. Ethical approval obtained from the participating hospitals and from The Nara Medical University. All participants provided written informed consent. One patient was lost during the follow-up period, and another one died of unrelated causes at one and half-year follow-up. Therefore, 71 hips (60 patients) were retrospectively followed-up for a minimum of 3 years (or until the conversion to total hip replacement). The patients included 45 men and 15 women. The patient average age at the time of surgery was 39 years (range, 15–61 years). Thirty-four patients had bilateral diseases, and 11 underwent bilateral free vascularized fibular grafting. The average interval between the first operated side and the second operated side was 9 months (range, 1–24 months). Six patients with an interval greater than half a year refused immediate second operation because of asymptomatic osteonecrosis. All hips were followed for a minimum of 3 years following free vascularized fibular grafting, and the average duration of follow-up was 7 years (range, 3–12 years). Etiologies were alcohol abuse in 31 hips (44%), steroid use in 27 (38%), idiopathic in 7 (10%) and trauma in 6 (8%). Steroid use, defined as an exposure to at least 16.6 mg/day predonisolone, was considered an etiologic factor [20].

**Figure 1:**
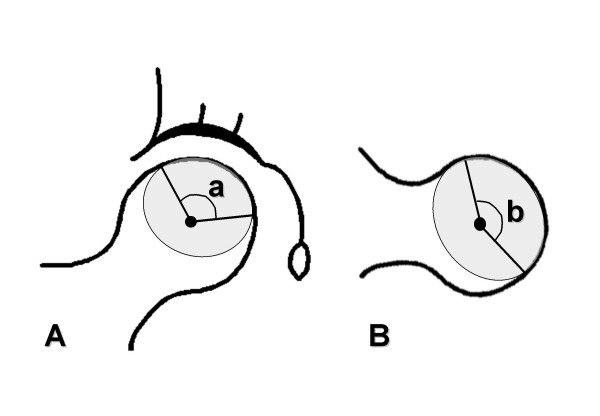
Diagrams showing the measurement of the extent of necrosis. **A **Anteroposterior angle. **B **Lateral angle. a plus b was considered as the extent of necrosis.

**Figure 2:**
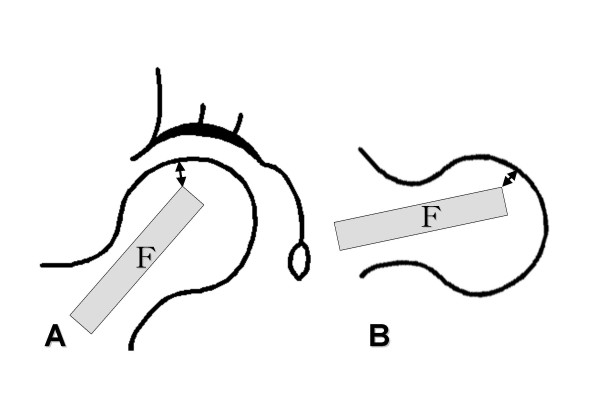
Diagrams showing the measurement of the distance from the tip of the grafted fibula to the subchondral bone of the femur head. **A **Anteroposterior radiograph **B **Lateral radiograph. The shorter distance was used for statistical analysis of the relationship between the distance from the tip of the grafted fibula and postoperative radiographic progression. F: fibula.

Preoperative staging of the necrotic lesion was performed using the Steinberg's classification system (Table 1) [21]. The extent of osteonecrosis was also measured on antero-posterior and lateral radiographs before surgery according to Kerboul et al (Figure 1) [22]. The distance from the tip of the grafted fibula to the subchondral bone of the femoral head was measured on antero-posterior and lateral radiographs immediately taken after surgery (Figure 2). The shorter distance was used for statistical analysis of the relationship between the distance from the tip of the grafted fibula and postoperative radiographic progression.

**Table 1:** Preoperative stages of the 71 hips classified according to the Steinberg's classification system.

Stage	Characteristics	Number of Hips
0	Normal radiographs, bone scan, and MR images	0
I	Normal radiographs, abnormal bone scan, and MR images	
A: mild	< 15% of head involvement	0
B: moderate	15% to 30%	2
C: severe	> 30%	1
II	Abnormal radiograph	
A: mild	< 15% of head involvement	1
B: moderate	15% to 30%	3
C: severe	> 30%	24
III	Subchondral collapse producing a crescent sign	
A: mild	< 15% of articular surface	0
B: moderate	Crescent beneath 15% to 30%	1
C: severe	Crescent beneath > 30%	2
IV		
A: mild	< 15% of surface collapsed and depression is < 2 mm	2
B: moderate	15% to 30% collapsed or 2 to 4 mm depression	8
C: severe	> 30% collapsed or > 4 mm depression	24
V	Joint narrowing with or without acetabular involvement	
A, B, or C	Average of femoral head involvement, as determined in Stage an d estimated acetabular involvement	3
VI	Advanced degenerative changes	0

At the time of surgery and at each follow-up examination, the Harris hip score [23] was used for clinical evaluation, and radiographic progression was evaluated. Radiographic progression was evaluated for assessing the development of necrotic lesion, changes in the contour of the femoral head, or progression of osteoarthritis. The development of necrotic lesion was measured on antero-posterior and lateral radiographs according to Kerboul et al's methods. The depth of depression was measured on the latest radiographs using plastic templates with 1-mm increments Mose concentric circles were compared with the radiographs before surgery. Progression of osteoarthritis was decided according to the narrowing of joint space or the formation of osteophytes. Outcome was graded as excellent when the Harris hip score was greater than 90 points, good when it was between 80 and 89 points, fair when it was between 70 and 79 points, and poor when it was less than 70 points, according to previous reports [14,17]. Conversion to total hip replacement was used as the end point in the present study.

### Surgical technique

**Figure 3:**
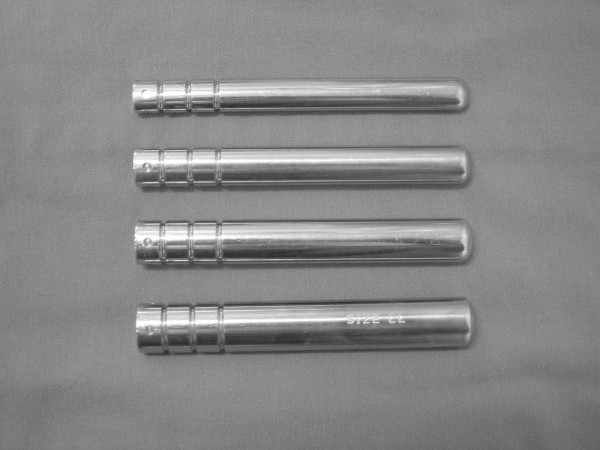
Metallic bars made mimicking the fibular shape were used for trials. There are 4 sizes: 12, 14, 16, 18 mm in width.

Surgery was performed with the patient in the supine position. The vascularized fibula was harvested from the ispilateral leg using a lateral approach as previously described by Urbaniak et al. [15]. The peroneal monitoring flap, which is supplied from the cutaneous perforator of the peroneal artery was made from the overlying skin of the fibula, and was used to monitor vascular patency of the grafted fibula after suturing the vessels. Then, a slightly curved (medial convex) skin incision of 10 cm was made in the inguinal area. The lateral femoral circumflex artery and the comitant veins were identified. Using a lateral approach in the proximal thigh, the lateral aspect of the proximal part of the femur was exposed through the separated tensor fasciae latae and the vastus lateralis. Under fluoroscopic control, a guide-pin was inserted from the subtrochanteric region into the necrotic lesion in the femoral head. A tunnel was created with reamers of gradually increasing sizes (8 to 19 mm in diameter). Using a high-speed burr (Cebatome, Zimmer, Warsaw IN) and a curette under fluoroscopic image control, remaining necrotic to subchondral bone was curetted as much as possible, and the tunnel was prepared. Successful tunnel preparation was confirmed with some trials using an imitated fibula (Figure 3). Then, the fibula was introduced through the tunnel, and was positioned beneath the subchondral bone of the femoral head within the cancellous bone chip harvested from the greater trochanter and the iliac crest. The fibula was stabilized to the femur with a cannulated titanium mini-screw or a Kirschner wire. Finally, the peroneal vascular bundle was introduced anteriorly, and arterial and venous anastomoses were performed under an operating microscope. Bleeding from the grafted fibula and monitoring of the buoy flap confirmed vascularization of the graft.

A posterior below-the-knee cast was applied to prevent hammer toes for 2 weeks, and the patient was instructed not to bear weights for 12 weeks. Then, a gradual weight-bearing program was initiated. Full weight-bearing was allowed approximately 6 months after surgery. Follow-up examinations with radiographs and clinical evaluation were performed every half a – year.

### Statistical analysis

A paired t-test was used for the statistical analysis of the relationship between preoperative Harris hip score and the latest score. The Chi-square for independence test was used for the statistical analysis of the relationships between the latest score and etiology, the latest score and stage, the latest score and extent of osteonecrosis, postoperative radiographic progression and etiology, postoperative radiographic progression and stage, postoperative radiographic progression and extent of osteonecrosis, survival and etiology, survival and stage, survival and extent of osteonecrosis. A Mann-Whitney's U test was used for the statistical analysis of the relationships between the distance from the tip of the grafted fibula and postoperative radiographic progression. The Kaplan-Meier test and 95% confidence interval were used for survival analysis. Statistical significance was set at p < 0.05. Power analysis was also performed.

## Results

### Clinical outcomes

The average preoperative Harris hip score was 56 points (range, 21–96 points), and the average score at the latest follow-up was 78 points (range, 26–100 points). The Harris hip score was determined for all hips at the final follow-up examination. The scores of 13 hips which had undergone total hip replacement were determined at the time just before total hip replacement. There were significant differences between the preoperative scores and the latest scores (p = 0.000000016). At the latest follow-up examination, 31 hips (44%) were rated excellent, 16 hips (23%) were rated good, 4 hips (6%) were rated fair, and 20 hips (28%) were rated poor. In 31 hips with alcoholic osteonecrosis, 24 hips (77%) were rated good to excellent. In 27 hips with steroid-induced osteonecrosis, only 14 hips (52%) were rated good to excellent. In 7 hips with idiopathic osteonecrosis, 5 hips were rated good to excellent. In 6 hips with traumatic osteonecrosis, 4 hips were rated good to excellent. There was a significant difference between steroid-induced osteonecrosis and alcoholic osteonecrosis (p = 0.038, statistical power = 0.51). The rate of good or excellent cases in steroid-induced osteonecrosis was worse than that in alcoholic osteonecrosis.

With regards to preoperative stage, in 31 hips without preoperative collapse (Steinberg's stages I and II), 27 hips (87%) were rated good to excellent. In 40 hips with preoperative collapse (Steinberg's stages III, IV and V), only 20 hips (50%) were rated good to excellent. Preoperative collapse was noted to be significantly associated with rate of good or excellent cases (p = 0.001, statistical power = 0.98). The rate of good or excellent cases in hips with preoperative collapse was worse than that in hips without collapse (Table 2).

**Table 2:** Results of Free Vascularized Fibula Grafting Using the Steinberg's Classification System.

Stage	Hips with Excellent or Good Score (> 70)	Radiographic Progression	Conversion to THA
IB (n = 2)	2 hips	0	0
IC (n = 1)	1 hip	1 hip	0
IIA (n = 1)	1 hip	0	0
IIB (n = 3)	3 hips	0	0
IIC (n = 24)	20 hips	9 hips	1 hip
IIIB (n = 1)	1 hip	0	0
IIIC (n = 2)	0	2 hips	2 hips
IVA (n = 2)	2 hips	0	0
IVB (n = 8)	3 hips	4 hips	3 hips
IVC (n = 24)	14 hips	16 hips	5 hips
VC (n = 3)	0	3 hips	2 hips

With regards to the extent of osteonecrosis, in 50 hips with less than a 300-degrees extent, 41 hips (82%) were rated good to excellent. In 21 hips with an extent greater than 300-degrees, only 7 hips (33%) were rated good to excellent. Preoperative extent of osteonecrosis was noted to be significantly associated with the latest score (p = 0.0001, statistical power = 0.98). The rate of good or excellent cases in hips with an extent greater than 300-degrees was worse than that in hips with less than a 300-degrees extent.

### Radiographic outcomes

At the latest follow-up examination, radiographic progression was noted in 35 hips (49%). In 27 hips with steroid-induced osteonecrosis, 15 hips (56%) have progressed. In 31 hips with alcoholic osteonecrosis, 16 hips (52%) have progressed. In 7 hips with idiopathic osteonecrosis, 3 hips have progressed. In 7 hips with traumatic osteonecrosis, one hip has progressed. There were no significant differences among etiologies (p = 0.38, statistical power = 0.06).

With regards to preoperative collapse, in 31 hips without preoperative collapse (Steinberg's stages I and II), 10 hips (32%) have progressed. In 40 hips with preoperative collapse (Steinberg's stages III, IV and V), 25 hips (63%) have progressed. Preoperative collapse was noted to be significantly associated with radiographic progression (p = 0.011, statistical power = 0.72). The rate of postoperative progression in hips with preoperative collapse was worse than that in hips without collapse (Table 2). When we divided Steinberg's stage IV into mild collapse (collapse less than 3 mm) and severe collapse (collapse more than 3 mm), following the Japanese Orthopaedics Association staging system [24], the rates of radiographic progression were 53% for hips with mild collapse (10 of 19 hips) and 73% for hips with severe collapse (11 of 15 hips). However, there was no statistical significant difference between mild collapse and severe collapse (p = 0.19, statistical power = 0.26). The difference might very well have been statistically significant if a larger number of hips had been included in the study.

With regards to the extent of osteonecrosis, in 50 hips with less than a 300-degrees extent, 20 hips (40%) have progressed. In 21 hips with an extent greater than 300-degrees, 15 hips (71%) have progressed. The extent of osteonecrosis was noted to be significantly associated with radiographic progression (p = 0.015, statistical power = 0.97).

### Survival rate

During the follow-up period, 13 hips in 11 patients received total hip replacement, with an overall survival rate of 83% with a 95% confidence interval (range, 74–92%) 7 years after surgery, when conversion to total hip replacement was considered as the endpoint. The average time to conversion to total hip replacement was 4 years (range, 1.5–10 years). In 27 hips with steroid-induced osteonecrosis, 18 hips survived. The survival rate was 68% with a 95% confidence interval (range, 49–87%) 7 years after surgery. In 31 hips with alcoholic osteonecrosis, 28 hips survived. The survival rate was 90% with a 95% confidence interval (range, 79–100%) 7 years after surgery. In 7 hips with idiopathic osteonecrosis, 6 hips survived. In 6 hips with traumatic osteonecrosis, all hips survived. There was a significant difference between steroid-induced osteonecrosis and alcoholic osteonecrosis (p = 0.032, statistical power = 1). The survival rate in steroid-induced osteonecrosis was worse than that in alcoholic osteonecrosis.

Preoperative collapse was also significantly associated with joint survival. Survival rate of Steinberg's stages III, IV, and V osteonecroses was 72% with a 95% confidence interval (range, 57–87%) 7 years after surgery, whereas that of Steinberg's stages I and II osteonecroses was 97% with a 95% confidence interval (range, 91–100%) 7 year after surgery (p = 0.002, statistical power = 0.71) (Table 2).

With regards to the extent of osteonecrosis, in 50 hips with a mild or moderate extent (less than 300 degrees), the survival rate was 88% with a 95% confidence interval (range, 81–99%) 7 years after surgery. In 21 hips with a severe extent (greater than 300 degrees), the survival rate was 67% with a 95% confidence interval (range, 43–86%) 7 years after surgery. There was a significant relationship between the extent of osteonecrosis and survival rate (p = 0.04, statistical power = 0.87).

### Distance

In 36 hips without postoperative radiographic progression, the average distance from the tip of the grafted fibula to the subchondral bone of the femoral head was 2.7 mm (range, 0–10 mm). In 35 hips with postoperative radiographic progression, the average distance was 2.9 mm (range, 0–8.5 mm). There was no relationship between the distance from the tip of the grafted fibula to the subchondral bone of the femoral head and postoperative radiographic progression.

### Complications

Nine of 71 hips required reexploration due to venous occlusions. Thrombectomy and/or reanastomosis to other vessels were performed and all cases were successfully salvaged by reoperation. Radiographic progression was noted in 7 hips, which was not significantly observed compared to 62 other patients (p = 0.069), but radiographic progression occurred in 78% of the cases. Three hips received total hip replacements in this group. Three subtrochanteric oblique fractures occurred from the core to the shaft as a result of a fall. All patients were treated with open reduction and internal fixation with external immobilization. No vascular damage was detected, and outcomes of free vascularized fibular grafting were excellent at the latest follow-up examination. Hammer toes were detected in 10 patients. Painful flexion contracture of the toes was treated by cutting the flexor hallucis longus muscle [25]. Although, the reason for a high rate of hammer toes was not clear, the long period (12 weeks) of non weight bearing was one of the reasons in the present study.

## Discussion

Many difficulties exist when comparing the outcomes of free vascularized fibular grafting from several previous reports because of differences in operative techniques, use of different staging systems, and different clinical score systems. In the current study, clinical outcomes of steroid-induced osteonecrosis contributed to a high rate of poor outcomes. Radiographic progression was observed in about half of the hips studied. There was no relationship the between distance from the tip of the grafted fibula to the subchondral bone of the femoral head and radiographic progression, whereas preoperative collapse and the extent of the disease commonly affected the radiographic outcomes. Survival rate was 83% seven years after surgery. Steroid, preoperative collapse, and severe extent of the disease were negatively correlated with survival rate.

Yoo et al. [14] reported that no significant relationship existed between clinical outcomes and etiology. Berend et al. noted that there were significant differences between outcomes of treatment and etiology in their report of post-collapse osteonecrosis with an average of 4.3-year follow-up [26]. They reported that hips with idiopathic and alcohol-related osteonecroses had a worse prognosis than those with corticosteroid-induced osteonecrosis. However, the current study showed that the clinical scores and survival rates in steroid-induced osteonecrosis were worse than those with other etiologies. Recently, Weinstein et al. [27] reported the apoptosis of osteocytes in the femora of patients with steroid-induced osteonecrosis of the hip, and Eberhardt et al. [28] observed trabecular bone matrix degeneration and osteocyte death in the femora of steroid-treated rabbits. These reports indicated a lesser viability of bone in steroid-induced patients, and possibly worse outcomes in steroid-induced cases. Outcomes in the current study supported these previous reports.

Many researchers stated that the preoperative stage corresponded to radiographic progression. Yoo et al. [14] reported a significant relationship between clinical outcomes and the Ficat's staging system [29]. Outcomes of Ficat's stage III (osteonecrosis with preoperative collapse) were worse than those of Ficat's stage II (osteonecrosis without preoperative collapse). The current study showed that the preoperative stage was significantly associated with the rate of clinical score, radiographic progression, and survival rate. All outcomes of osteonecrosis with preoperative collapse were worse than those of osteonecrosis without preoperative collapse. Sotereanos et al. stated that the inevitability of progressive collapse of the femoral head after development of a crescent sign or progressive osteoarthrosis after collapse had been established long ago [17]. However, Magnussen et al. investigated articular cartilage degeneration in post-collapse osteonecrosis mechanically, histologically, and macroscopically [30]. They reported that there was no significant difference between Marcus's stage IV (flattening of the femoral head without signs of cartilage degeneration) and stage V (joint narrowing with or without acetabular involvement) [31]. They suggested that femoral head-sparing surgical strategies designed to restore the contour of the femoral head might be used in some patients with advanced osteonecrosis disease. When we divided Steinberg's stage IV into mild collapse and severe collapse, the rates of radiographic progression were 53% for hips with mild collapse and 73% for hips with severe collapse. Restoring severe collapse cases is more difficult than mild collapse cases. The difference might very well statistically significant if a larger number of hips had been included.

Sotereanos et al. reported that the stages in which femoral head involvement was more than 30% had a high probability of conversion to total hip replacement [17]. In the current study, the extent of osteonecrosis was associated with the clinical score, radiographic progression and survival rate. Although various factors help surgeons and patients to decide whether to convert to total hip replacement, a severe extent of osteonecrosis greater than 300 degrees was one of the important factors influencing the outcomes of free vascularized fibular grafting.

Thirteen hips received total hip replacement in the current study. Twelve of 27 hips (44%) that had 2 or 3 negative factors including steroid use or preoperative collapse or severe extent of osteonecrosis more than 300 degrees, received total hip replacement. Only one of 44 hips that had no or one negative factor had a total hip replacement. Ohzono et al. reported the natural history of nonoperative hips, and stated the necessity of operative treatment for moderate or large size osteonecrosis [2]. When considering operative treatment, core decompression is less successful for hips that have already collapsed [7]. Osteotomies are effective for hips with preoperative collapse and moderate size osteonecrosis, but are difficult for large osteonecrosis [8,9]. In our study, hips with moderate or large size osteonecrosis involving less than 300 degrees of the femoral head without preoperative collapse, were good candidates for free vascularized fibular grafting. Some researchers recently reported good results of rotational osteotomies combined with vascularized bone graft for advanced large size osteonecrosis, but the results of long-term follow-up are not known [32,33]. Hips with severe preoperative collapse and a large extent of osteonecrosis require hip replacements for the present.

## Conclusion

In conclusion, steroid-induced osteonecrosis was associated with poor scores and survival rate. Preoperative collapse was associated with poor scores, radiographic progression and poor survival rate. The large extent of osteonecrosis involving more than 300 degrees of the femoral head was associated with poor scores, radiographic progression and poor survival rate. The radiographic results were worse with severe collapse (greater than 3 mm) compared to mild collapse with poor results found in 73% and 53% of the cases, respectively. Small osteonecrosis (less than 300 degrees of the femoral head) without preoperative collapse (Steinberg's stages I and II) is a major indication for free vascularized fibular grafting while steroid-induced osteonecrosis is relative contraindication, and large osteonecrosis (greater than 300 degrees) with severe preoperative collapse is a major contraindication. Complications were relatively high in the current study. Free vascularized fibula grafting is a technically difficult procedure and should not be performed by those who have not had experience in microvascular techniques and with this procedure in particular. Hips with 2 negative factors such as severe preoperative collapse and a large extent of osteonecrosis, require hip replacements.

## Competing interests

The author(s) declare that they have no competing interests.

## Authors' contributions

KK, HY, HO, TO, YK KM KS, KK, IK, NM, KT and ST participated in the operations. KK, KS and YT participated in the design of the study and performed the statistical analysis. KK, HY and KM drafted the manuscript. All authors read and approved the final manuscript.

## Pre-publication history

The pre-publication history for this paper can be accessed here:


